# Distribution and risk factors associated with intestinal parasite infections among children with gastrointestinal disorders

**Published:** 2016-12

**Authors:** Hamed Kiani, Ali Haghighi, Roya Salehi, Eznollah Azargashb

**Affiliations:** 1 *Department of Medical Parasitology and Mycology, School of Medicine, Shahid Beheshti University of Medical Sciences, Tehran, Iran*; 2 *Iranian Veterinary Organization. Hamadan, Iran*; 3 *Department of Community Medicine, School of Medicine, Shahid Beheshti University of Medical Sciences, Tehran, Iran*

**Keywords:** Parasitic infection, Distribution and risk factors, Children, Western Iran

## Abstract

**Aim:**

Prevalence and risk factors associated with intestinal parasites among children ≤ 12 years old in Nahavand county western Iran, was the objective of this search.

**Background:**

Intestinal parasites (IPs) are important health problems among most societies.

**Methods:**

This cross sectional study was carried out during 6 months from April to September 2014 in Nahavand County western Iran. Fecal samples were collected from 500 children suffering from gastrointestinal disorders (GIDs) and examined by macroscopy and microscopic (using saline and iodine wet mount, formalin-ether sedimentation, Trichrome and modified Ziehl Neelsen staining) methods. Finally, data was analyzed using Chi-square (Chi2) test and Fisher’s exact test as well as logistic regression.

**Results:**

21.8% (109/500) of the samples were infected by one or more IPs. The most common parasites were *Blastocystis *sp. (16.2%), followed by *Cryptosporidium *spp. (2.6%), *Giardia lamblia *(1.6%), and *Entamoeba coli *(1.6%). Prevalence of intestinal parasite infections were significantly associated with age (OR= 2.280; CI 95% = 1.375-3.830; P<0.002), gender (OR= 0551; CI 95% = 0.348-0.875; P<0.011), contact with domestic animal or soil (OR= 0.492; CI 95% = 0.282-0.860; P<0.013) and seasons (OR= 2.012; CI 95% = 1.254-3.227; P<0.004). There was a significant correlation between IPs with diarrhea (OR= 3.027; CI 95% = 1.712-5.345; P<0.001) and nausea or vomiting (OR= 3.261; CI 95% = 1.281-8.175; P<0.013).

**Conclusion:**

*Blastocystis *sp. was the most prevalent parasites among children in Nahavand County and Helminthes infection have been dramatically decreased. Our finding shown that gender, age, season and contact with domestic animals or soil polluted are main predictive factors for intestinal parasite infections among children in this region. Moreover, IPs infection among children with gastrointestinal disorders were significantly associated with diarrhea and vomiting or nausea signs.

## Introduction

Intestinal parasitic infections (IPIs) can cause acute gastrointestinal disorders (AGIDs), and these organisms result in most common communicable diseases (1-3). Human parasitic infections lead to significant morbidity and mortality among different societies in the world (4,5). Globally, more than 3.5 billion individuals are infected by IPs, and of them 450 million are ill as a result of IPs, the main part of them are being children (6-8). Protozoan infections such as (Ameobiasis, Giardiasis and Cryptosporidiosis) and helminthic infections such as (Ascariasis, Enterobiasis, hookworms, and Trichuriasis) are among the most common intestinal parasitic infection in the worldwide. These organisms are also known as serious public health problems in children and complications such as iron deficiency (anemia), vitamin A deficiency, diarrheal or dysentery, malnutrition, delay growth, physical and mental health problems (7- 13). Moreover, IPIs can result in serious problems in gastrointestinal disorders patients, immunocompromised patients such as HIV positive, transplanted and hemodialysis patients (2, 14, 15). The incidence and frequency rate of IPIs vary in different countries due to environmental condition, geographical factors, location, level of family education, a range of family income, health education, accessibility of threatened/or unthreatened water, hand washing practices before meal and after the toilet and etc (16-19). The epidemiological data based on the frequency of IPIs is one of the basics for developing appropriate control measures. Previous epidemiological studies in Iran have demonstrated high prevalence of IPIs among different population and have shown that *Blastocystis *sp., *Entamoeba histolitica/E. dispar*, *Giardia lamblia*, *Cryptosporidium spp*., *Enterobius vermicularis *and *Ascaris lumbericoides *are the predominant IPIs (1, 2, 15, 20-25). Surprisingly, in spite of the public health importance of these infections and the potential consequences, there was no enough data about their epidemiology of IPIs among children in western Iran. The aims of this study were to identify the epidemiology and associated risk factors of IPIs in children with gastrointestinal disorders in Nahavand County, western Iran.

## Materials and Methods

**Study areas & population:**

This cross-sectional survey was carried out from April to September 2014 in Nahavand County, Hamadan province, western Iran. Stool samples from 500 children under 12 years old suffered from GIDs who referred to medical centers laboratories were randomly collected. The sample size was calculated using formula: n = z2*p(1-p)/d2 under assumptions as follow: the reference prevalence was considered as 35 % (20), a 95% level of confidence and 5% marginal error. The sample size required was 496, but for more accuracy 500 samples were collected.

**Questionnaire**

The questionnaire was prepared to elicited information on the demographic data (age, gender and location (urban

& rural)), acute clinical symptoms (diarrhea and dysentery, abdominal pain, stomach pain, bloating, vomiting & nausea), environmental sanitation and living condition characteristics (season, contact with domestic animals and soil) which will be used to assess the potential risk factors for IPIs. Informed consent was obtained from each attendee prior to the sampling. Certain criteria were applied to enroll children in the study. Inclusion criteria included age ≤12 years old with no history of taken anti parasites drugs in two weeks before the test. Exclusion criteria included children suffering from GIDs (diarrhea, dysentery diarrhea, stomach pain, bloating and Nausea or vomiting) disease.

**Stool Collection and Processing**

From all of the participants a single fresh stool sample were collected from 12 clinical laboratories in Nahavand County. The collected specimens were transported to the parasitological laboratory of Ayatollah Alimoradian hospital, in Nahavand city for the stool analysis process. At first, Information related to patients was recorded on a daily basis in the registered office information and stool samples were observed macroscopically to consistency and the presence of worm, larva, segments, blood, and mucus were recorded. After macroscopic analysis, direct wet mount with normal saline (0.85% NaCl solution) in all of the stool specimens in one side of the slides (for the presence of motile intestinal parasites and trophozoite) was done and lugol’s iodine staining was performed to distinguish cysts of intestinal parasites in other side of the slide. In the next step formalin- ether concentration was prepared and the sediments were stained with iodine, put on a slide and covered with a cover slip to accurate detection of cyst or eggs of intestinal parasites. Moreover, slide smear was prepared in fresh stool and immediately stained with trichrome’s staining to accurate differentiation of intestinal protozoa *(Entamoeba*, *Giardia*, *Blastocystis *sp. and etc). For detection of coccidian parasites (*Cryptosporidium *spp.), Modified Zeihl- Neelsen staining after concentration technique was done. All of the smears prepared were microscopically observed using 100x, 400x, and1000 x magnification (26).

**Statistical analysis**

For statistical analysis the data was exported to Statistical Package for the Social Sciences software version16 (SPSS, Chicago, IL, USA). The proportion percentage was used to describe the characteristics of the participants, including the frequency of IPIs according to age, sex and etc. A Pearson’s Chi-square (Chi2) test and Fisher’s exact test were used for differences in the proportions of IPIs between different variables and odds ratios (OR) and 95% confidence intervals (CI) were used for associations of variables. The t-test wasused to compare the mean age. Logistic regression was used to detect risk factors of parasite infection. The *P *value <0.05 was considered statistically significant.

**Ethical clearance**

The all the procedures of this study were approved by the Ethics Committee of the Shahid Beheshti University of Medical Science (SBMU). Iran, before the beginning of the study. All study participants were informed about the study procedures and written informed consents were obtained from all of them prior to sample collection.

## Results

**Sociodemographic characteristic**

Out of 500 children with GIDs, 278 boys and 222 girls, aged 22 days-12 years were enrolled in this study. 246 children lived in rural areas and 254 subjects lived in urban area.149 of 500 participants had frequently contact with domestic animal or soil and 351 child no contact. The proportion of children according to age groups were ≤ 3years (276 patients), 4 – 6 years (135 children) and 7 – 12 years (89 isolates). These specimens were taken during spring (224 patients) and summer season (276 patients). According to macroscopic analysis and consistency of stools 272 (54.4%) child have diarrheal stool and frequency of patients with formed, soft, dysenteric diarrhea and hard stools was 145 (29%), 49 (9.8%), 11 (2.2%) and (4.6%) respectively.

**Table 1:** Frequency of IPIs and poly-parasitism among children with GIDs in Nahavand County, western Iran (N = 500).

Type of parasites	Mono parasites n (%)	Mixed infections n ( %)	Total n (%)
Total infected patients	97 (19.4)	12 (2.4)	109 (21.8)
Protozoa			
*Blastocystis *sp*.*	70 (14)	11 (2.2)	81 (16.2)
*Giardia lamblia*	7 (1.4)	1 (0.2)	8 (1.6)
*Cryptosporidium *spp.	7 (1.4)	6 (1.2)	13 (2.6)
*Entamoeba histolytica/E. dispar*	0	1 (02)	1 (0.2)
*Entamoeba coli*	4 (0.8)	4 (0.8)	8 (1.6)
*Endolimax nana*	1 (0.2)	1 (0.2)	2 (0.4)
*Iodamoeba * *bucheli*	1 (0.2)	0	1 (0.2)
*Entamoeba hartmanni*	3 (0.6)	0	3 (0.6)
*Trichomonas hominis*	2 (0.4)	0	2 (0.4)
*Chilomastix mesnilii*	1 (0.2)	1 (0.2)	2 (0.4)
Helminthes			
*Enterobius vermicularis*	1 (0.2)	0	1 (0.2)
Children infected with protozoa	96 (19.2)	12 (2.4)	108 (21.6) *
Children infected with helminthes	1 (0.2)	0	1 (0.2)

n: number;

* The frequency of intestinal protozoa was significantly higher than intestinal helminthes.

**Table 2:** Frequency of IPIs by socio-demographic and clinical features in children with GIDs in Nahavand County, western Iran (n = 500).

Variables	Positive n %	Negative n %	Totl n %	OR	CI 95%		*P-value*
					*Lower*	*Upper*	
Gender							0.011
Male	71 (25.5)	207 (74.5)	278 (100)	Reference			
Female	38 (17.1)	184 (82.9)	222 (100)	0.551	0.348	0.873	
Age (Year)							0.008
≤3	44 (15.9)	232 (84.1)	276 (100)	Reference			
4-6	44 (32.6)	91 (67.4)	135 (100)	2.280	1.357	3.830	0.002
7-12	21 (23.6)	68 (76.4)	89 (100)	1.484	0.800	2.755	0.211
Residence							0.606
Urban	51 (20.1)	203 (79.9)	254 (100)	Reference			
Rural	58 (23.6)	188 (76.4)	246 (100)	1.147	0.680	1.936	
Contact with domestic animal & soil							0.013
Yes	49 (32.9)	100 (67.1)	149 (100)	Reference			
No	60 (17.1)	291 (82.9)	351 (100)	0.492	0.282	0.860	
Seasons							0.004
Spring	35 (15.6)	189 (84.4)	224 (100)	Reference			
Summer	74 (26.8)	202 (73.2)	276 (100)	2.012	1.254	3.227	

n: number; OR: odds ratio; Reference: The top group is considered as baseline.

* Chi 2 test and univariate Logistic regression was used

**Table 3:** Clinical features associated with IPIs among children with GIDs in Nahavand County, western Iran (n = 500).

Characteristics	Total examined	Infected N (%)	Non infected N (%)	OR	CI _95%_		*P. *value
					*Lower*	*Uper*	
Abdominal pain							0.371
Yes	368	79 (21.5)	289 (78.5)	Reference			
No	132	30 (22.7)	102(77.3)	1.265	0.756	2.119	
Nausea or vomiting							0.013
Yes	21	9 (42.9)	12(57.1)	3.236	1.281	8.175	
No	479	100 (20.9)	379(79.9)	Reference			
Stomach pain							0.041
Yes	185	30 (16.2)	155(83.8)	0.586	0.352	0.979	
No Bloating	315	78 (24.8)	237(75.2)	Reference			0.241
Yes	57	13 (22.8)	44(77.2)	1.522	0.755	3.067	
No	443	96 (21.7)	347(78.3)	Reference			
Type of stool							0.002
Formed stool	145	17 (11.7)	128 (88.3)	Reference			
Soft stool	49	10 (20.4)	39 (79.6)	1.931	0.817	4.560	0.134
Diarrheal stool	272	78 (28.7)	149(71.3)	3.027	1.712	5.354	0.001
Dysenteric diarrhea stool	11	2 (18.2)	9 (81.8)	1.673	0.333	8.400	0.532
Hard stool	23	2 (8.7)	21 (91.3)	0.717	0.154	3.332	0.671

OR: odds ratio; n: Number; Reference: The top group is considered as baseline;

* Chi 2 test as well as Fisher exact test and univariate Logistic regression for type of stool analysis are used.

**Distribution of Intestinal Parasitic Infections**

According to this study, 21.8 % (109/500) of the children were infected with one or more IPs infection, among them 97 (19.4%) and 12 (2.4%) of individuals had mono and poly parasitism respectively. The most common IPs among children was *Blastocystis sp. *(81 case/16.2%), followed by *Cryptosporidium spp. *(13 case/2.6%), *Giardia lamblia* (8 case/1.6%) and *Entamoeba coli *(8 case/1.6%). The prevalence of other IPs are shown in (Table 1). Among the all children 21.6% (108/500) were infected with protozoan parasites and 0.2% (1/500) were infected with helminthes than one parasite. More frequency of co-infection was *Criptosporidium *spp*. *with *Blastocystis *sp*. *in 1 % (5/500 cases) and *Entamoeba coli *with *Blastocystis *sp. In 0.8% (4/500). Other co-infection was *Entamoeba histolytica/E.* *dispar *with *Chilomastix mesnili *and *Giardia lamblia* with *Blastocystis *sp. Triple infection was also observed in one cases *Blastocystis hominis *with *Endolimax nana*

*& Cryptosporidium *spp. According to this results high frequency of poly parasitic infections observed in *Blastocystis* sp. 11 case (2.2%) and *Cryptosporidium *spp. 6 case (1.2%).

**Risk factors and Clinical features associated with (IPIs)**

Logistic regression by forward method detected the risk factors associated with IPIs among children with GIDs and socio-demographic, environmental and personal hygiene factors evaluated in this study(Table 2). Prevalence of IPIs had significantly different by gender (girls 17.1% was infection. Frequency of intestinal helminthes among this lower than boys 25.5%), (OR= 0.551; CI = 0.348-0.875;children was significantly lower than intestinal protozoa (P<0.011), age groups (children 4-6 years 32.6% higher < 0.001). 

**Table 4:** Frequency of *Cryptosporidium *spp., *Giardia lamblia *and *Blastocystis *sp*. *by socio-demographic and clinical features in children with GIDs in Nahavand County, western Iran (n = 500).

	Parasites									
Variables	*Cryptosporidium *spp.				*Giardia lamblia*			*Blastocystis *sp*.*		
	N*Total*	Positive (%)	Negative (%)	*P-value*	Positive (%)	Negative (%)	*P-value*	Positive (%)	Negative (%)	*P-value*
Age (Year)				0.023*			0.001*			0.009*
≤3	276	4 ( 1.44)	272(98.55)		0	276 (100)		35 (12.68)	241 (87.31)	
4-6	135	3 (2.22)	132 ( 97.77)		5 (3.7)	130 (96.3)		33 (24.44)	102 (75.55)	
7-12	89	6 (6.74)	83 (93.25)		3 (3.37)	86 (96.62)		13 (14.6)	76 ( 85.39)	
Location				0.021*			0.170			0.194
Rural	246	11 (4.47)	235 (95.52)		6 (2.439)	240 (97.56)		34 (13.82)	212(88.17)	
Urban	254	2 (0.787)	252 (99.21)		2 (0.787)	252 (99.21)		47 (18.50)	207(81.49)	
Contact with domestic …				0.001*			0.010*			0.155
Yes	149	12 (8.05)	137 (91.94)		6 ( 4.026)	143 ( 95.97)		30 (20.13)	119 (97.86)	
No	351	1 ( 0.285)	350 (99.71)		2 (0.569)	349 (99.43)		51 (14.52)	300 (85.47)	
Seasons				0.045*			0.080			0.017*
Spring	224	2 (0.892)	222 (99.1)		1 (0.446)	223 (99.55)		26 (11.6)	198 (88.39)	
Summer	276	11(3.98)	265 (96.01)		7 (2.53)	269 (97.46)		55 (19.92)	221(80.08)	
Vomiting & nausea				0.001*			1.000			0.506
Yes	21	8 (38)	13 (62)		0	21 (100)		5 (23.8)	16	
No	479	5 (1.045)	474(98.95)		8 (1.67)	471 (98.33)		75 (15.65)	403	
Diarrhea				0.031*			0.725			0.007*
Yes	256	11(4.29)	245 (95.7)		5 (1.95)	251 (98.04)		53 (20.7)	203 (79.29)	
No	244	2 (0.81)	242 (99.18)		3 (1.22)	241 (98.77)		28 (11.47)	216 (8.52)	

OR: odds ratio; n: Number; Reference: The top group is considered as baseline;

* Chi 2 as well as Fisher exact test are used. Also, univariate Logistic regression was used for age group analysis

## Discussion

Epidemiological studies based on frequency of IPs infection in different society, have primary objective to identify high-risk groups and formulate appropriate interventions. The present study attempted to assess the accurate data about the prevalence rate, associated risk factors and symptoms results from IPs among children with GIDs.

One hundred and nine patients (21.8%) were infected with IPs. This results is equal to previous study among patients ≥15 years old (24.3%) (21), and is very higher than checkup individuals under 15 years old (9.3%) in Nahavand laboratories (21). 21.6% of children infected by one or more protozoan parasites and only one children infected by helminthes (*Enterobius vermicularis*) infection. However prevalence of helminthes infection may be increased if cellophane tape, The Baerman techniques and agar plate culture were used (27). However, our study and recently reported studies in different part of Iran (28), Isfahan (29), Hamadan (30), Nahavand (21) represent a dramatic decrease of helminthic infections (*Ascariasis*, hook worms, *Entrobiosis *and etc.) and protozoan infections (Ameobiasis, Giardiasis, Cryptosporidiosis and etc.). It may be results from increasing public and individual hygienic, increase awareness of the prevention and control of parasitic infections and access to the safe food and water sources. Also, variations in prevalence of IPs may be due to differences in climatic conditions, environmental hygiene, economic and educational status and study subjects, and previous control efforts.

The most common IPs in our study was *Blastocystis *sp. with 81 cases (16.2%). It is similar to previous studied in Nahavand County. This study and other study in Tehran and Isfahan show that prevalence of *Blastocystis *sp. recently increased in Iran (21, 29, 31). But the pathogenicity of *Blastocystis *sp*. *is still controversial, some studies suggest that it is a pathogen (32, 33), whereas other studies believe it is a non-pathogen or commensal (33, 34). Recently, discussion on pathogenesis and accurate diagnosis from *Blastocystis *sp. can be cause of increase reported of this parasite. In our study *Blastocystis* sp. are related with diarrhea and season. Also previous clinical studies by Tan et al. and Moosavi et al. presented that abdominal pain and diarrhea are two the major signs among *Blastocystis*-positive patients (31,32). Regardless of *Blastocystis *sp., *Cryptosporidium *spp. *and Giardia lamblia* with the frequency of 13 (2.6%) and 8 (1.6%) respectivelly was the most common intestinal pathogenic protozoan. Because, staining method for *Cryptosporidium *spp. and concenteration methods are not usulally use in laboratories , Modified Zeihl-Neelsen staining after concentration technique specifically in children with GIDs is required in this region and other same regions.

In this study, *Cryptosporidium *spp*., Giardia lamblia *and *Blastocystis *sp. have the most prevalent IPIs in children with GIDs. Frequency of *Cryptosporidium *spp., *Giardia lamblia* and *Blastocystis *sp*. *with Socio-demographic and clinical features in children with GIDs are shown in Table 4. There was significant relationship between cryptosporidiosis by age (p = 0.023), location (p= 0.021), contact with domestic animal and soil polluted by animal feces (p= 0.001), season (p= 0.045), vomiting/ nausea (p= 0.001) and diarrhea (p= 0.031). Significantly different observed among Giardiasis by age (p= 0.001) and contact with domestic animal or soil (p= 0.010).There was significant correlation among Blastocystis sp. by age (p= 0.009), season (p= 0.017) and diarrhea sign (p= 0.007).

*Blastocystis *sp.,*Cryptosporidium *spp*. *and *Giardia lamblia* are zoonotic protozoa that are a great threat to public health and can be transmitted through contaminated water and food. Water and food contaminated with animal wastes and farming practice are important source of transmission. Intestinal parasitic infections, especially *Cryptosporidium *spp. and *Giardia lamblia *were significantly associated with contact with domestic animals and soil. Thus it is necessary that animals be kept away from water and food source that are used by human. Animal husbandry and agriculture are common in Nahavand County, in the warm season livestock going to pastures and contamination of water sources and vegetables by livestock fecal can increase pollution. Also frequency of IPs (especially *Cryptosporidium *spp. and *Blastocystis *sp.) in warm season (summer) is higher than cold season (spring).

According to age groups significant different was observed among children 6-12 years old by Cryptosporidiosis and giardiasis. It may be due to contact children with polluted source such as vegetable in farms or soil polluted by animal feces during play in soil land and farms especially in rural region or direct contact (person to person) during meal food or etc. together.

In relation to poly parasitism, our results are similar to previous study in Isfahan, Hamadan and previous study in Nahavand County that poly parasitism detected. The observed co-infection could be clarified by the facts that many species of protozoa (*Cryptosporidium *spp., *E. histolytica/E.* *dispar, Blastosystis *sp*., Entamoeba coli, Giardia lamblia *and etc*.*) have the same mode of transmission and that hygiene is poor in these areas (21,29,30).

Infection happen by IPs may be consequences such as gastrointestinal disorders (diarrhea or dysentery and etc., anemia, malabsorption, delay growth in children and physical complications, therefore, it was seen as the main health problem (31,35,36). According to clinical signs in our study there was significant correlation among IPs and cryptosporidiosis in GIDs children with diarrhea, and vomiting/ or nausea. Similar to our results various studies in other countries and in severe acute malnutrition related diarrhoea have shown intestinal parasites as risk factors of diarrhoea (37,38). Our results show that there are significant relationship between *Blastocystis *sp. by diarrhea sign and in previous study in Iranian patients moosavi et al.( *Blastocystis* sp), keshavarz et al. (*Cryptosporidium *spp.) and haghighi et al. ( *Entamoeba histolitica/E. dispar*) are significantly associated with diarrhea (2, 31 ,39). Also in turkey the most common complaint related with IPIs was intestinal dysmotility, nausea/ vomiting and abdominal distention (8). There were some limitations in our study.

1. Stool samples were collected only once, It is likely that the analysis of three consecutive stool samples could have increased the diagnostic sensitivity.
2. Sample collected was done during two warm season and there is no data about autumn and winter.
3. Due to the lack of facilities and financial funding we could not use all the laboratory available techniques, such as the Baerman technique to find larvae of helminth including *Strongyloides stercoralis *or cellophane tape to detect *Enterobius vermicularies *and agar plate culture to find accurate detection of helminthes larva.
4. Also, it is likely that the prevalence of some protozoa such as *Entamoeba *and *Blastocystis *sp. could be even higher if culture techniques had been used.

Based on the present study *Blastocystis *sp. is the prevalent parasites in Nahavand County. Especially pathogenic parasites such as *Cryptosporidium *spp. and *Giardia lamblia* among children with GIDs, still are prevalent in western Iran, and helminthes infection have been dramatically decreased during the past decades. Thus, Modified Zeihl-Neelsen staining after concentration technique is recommended for *Cryptosporidium *spp. identification in this area. Also, our finding suggests the risk factors such as gender, season and contact with domestic animals and soil polluted are increase susceptibility of children with IPs infection. IPs infection among children with GIDs are associated with diarrhea and vomiting/or nausea in this area and the role of parasites in children’s should be considered. Thus, health interventions to children and prevent children from contact with contaminated resources such as land and livestock waste in this area is necessary.
